# Effects of Modified Magnetite Nanoparticles on Bacterial Cells and Enzyme Reactions

**DOI:** 10.3390/nano10081499

**Published:** 2020-07-30

**Authors:** Lyubov S. Bondarenko, Ekaterina S. Kovel, Kamila A. Kydralieva, Gulzhian I. Dzhardimalieva, Erzsébet Illés, Etelka Tombácz, Arina G. Kicheeva, Nadezhda S. Kudryasheva

**Affiliations:** 1 Moscow Aviation Institute (National Research University), 125993 Moscow, Russia; l.s.bondarenko92@gmail.com (L.S.B.); KydralievaKA@mai.ru (K.A.K.); dzhardim@icp.ac.ru (G.I.D.); 2 Institute of Physics SB RAS, FRC KSC SB RAS, 660036 Krasnoyarsk, Russia; 3 Institute of Biophysics SB RAS, FRC KSC SB RAS, 660036 Krasnoyarsk, Russia; n-qdr@yandex.ru; 4 Institute of Problems of Chemical Physics RAS, 142432 Chernogolovka, Moscow Region, Russia; 5 University of Szeged, H-6720 Szeged, Hungary; Illes.Erzsebet@chem.u-szeged.hu (E.I.); E.Tombacz@chem.u-szeged.hu (E.T.); 6 Siberian Federal University, 660041 Krasnoyarsk, Russia; khyzylsyg@mail.ru

**Keywords:** magnetite nanoparticles, humic acids-coated magnetite nanoparticles, silica-coated magnetite nanoparticles, zeta potential, hydrodynamic diameter, toxicity, bioluminescence, bacterial assay, enzymatic assay, oxidative stress, *Photobacterium phosphoreum*, NADH:FMN-oxidoreductase, luciferase

## Abstract

Current paper presents biological effects of magnetite nanoparticles (MNPs). Relations of MNP’ characteristics (zeta-potential and hydrodynamic diameters) with effects on bacteria and their enzymatic reactions were the main focus. *Photobacterium phosphoreum* and bacterial enzymatic reactions were chosen as bioassays. Three types of MNPs were under study: bare Fe_3_O_4_, Fe_3_O_4_ modified with 3-aminopropyltriethoxysilane (Fe_3_O_4_/APTES), and humic acids (Fe_3_O_4_/HA). Effects of the MNPs were studied at a low concentration range (< 2 mg/L) and attributed to availability and oxidative activity of Fe^3+^, high negative surface charge, and low hydrodynamic diameter of Fe_3_O_4_/HA, as well as higher Fe^3+^ content in suspensions of Fe_3_O_4_/HA. Low-concentration suspensions of bare Fe_3_O_4_ provided inhibitory effects in both bacterial and enzymatic bioassays, whereas the MNPs with modified surface (Fe_3_O_4_/APTES and Fe_3_O_4_/HA) did not affect the enzymatic activity. Under oxidative stress (i.e., in the solutions of model oxidizer, 1,4-benzoquinone), MNPs did not reveal antioxidant activity, moreover, Fe_3_O_4_/HA demonstrated additional inhibitory activity. The study contributes to the deeper understanding of a role of humic substances and silica in biogeochemical cycling of iron. Bioluminescence assays, cellular and enzymatic, can serve as convenient tools to evaluate bioavailability of Fe^3+^ in natural dispersions of iron-containing nanoparticles, e.g., magnetite, ferrihydrite, etc.

## 1. Introduction

Magnetite nanoparticles (MNPs) have been widely used as adsorbents to extract ecotoxicants from waste and natural waters [1,2,3]. Thus, iron oxide nanomaterials, including magnetite (Fe_3_O_4_) can be found in engineered and natural aqueous environments due to their numerous applications. Hence, fate of these particles in aquatic systems is an issue of a considerable concern. However, given that bare MNPs are susceptible to air oxidation [4] and are easily aggregated in aqueous systems, the stabilization of the iron oxide particles is mandatory. Functionalization of MNP surfaces is usually desired, and various molecules can be used as capping agents. Humic acids (HA) being natural high-molecular compound, have a high affinity to Fe_3_O_4_ nanoparticles; the sorption of HA on the Fe_3_O_4_ nanoparticles enhances the stability of the nanodispersions by preventing their aggregation [5,6,7,8]. Previously [8,9], magnetite Fe_3_O_4_ nanoparticles coated with HA were used for the extraction of heavy metal ions from water. Under certain conditions, these insoluble magnetic materials get an advantage of being easily removed with magnetic field.

Numerous studies reported a successful functionalization of Fe_3_O_4_ nanoparticles with active molecules such as alkoxysilanes [10,11,12,13,14]. It was also shown that alkoxysilanes and MNPs are interconnected by strong covalent bonds. A functional molecule like alkoxysilane consists of at least two functional groups, where the first group is applied for the attachment to the MNP surface, while the second group provides an adhesion point to the molecule bond [15]. The Fe_3_O_4_ nanoparticles grafted with SiO_2_ and alkoxysilanes are promising nanobiotechnological reagents due to their biocompatibility and hydrophilic properties. A huge variety of alkoxysilanes allows for the different types of surface functionalization by introducing charges into the system. As a result, the functionalization prevents the nanoparticle’ aggregation in liquids and improves their chemical stability [16]. Various applications of magnetite-alkoxysilane nanoparticles are known, such as detoxifying agents in ecological control [17], for drug delivery systems in medicine [18], technical use as thermal insulators [19], for soil stabilization [20,21,22], and in significant soil improvement [23]. It has been reported that SiO_2_-based nanoparticles could be used as fertilizers to enhance a seed germination and stimulate antioxidant system of crops [24]; they could also be used for pesticide delivery [25] and increasing the SiO_2_ entering into the terrestrial system [26].

A wide application range of MNPs encourages the deeper understanding of their biological activity. Since modified MNPs are potential adsorbents, their effects on water microorganisms are of special interest. Effects of modified MNPs on aqueous microorganisms are currently under study; a series of agents, such as anchored SnO_2_ [27], TiO_2_ [28], chitosan [29], Ag [30], oleic acid [31], and glycine [32,33] were previously used to modify an MNP surface. Oleic acid and glycine modifications showed lower cell toxicity as compared to bare nanoparticles, whereas TiO_2_ revealed a higher antibacterial activity.

In this study, we chose luminous marine bacterium as a model microorganism to evaluate and compare biological effects of three types of magnetite nanoparticles: bare MNPs, modified by humic acids, and silica (3-aminopropyltriethoxysilane, APTES), i.e., Fe_3_O_4_, Fe_3_O_4_/HA, and Fe_3_O_4_/APTES, respectively.

The bioluminescence bacteria-based assay is a traditional toxicity analysis; it has been applied for more than 50 years for toxicity monitoring due to its high sensitivity [34,35,36,37,38,39]. This bioassay uses luminescence intensity as a physiological test parameter. The registration of bioluminescence provides a convenient approach with high rates, low costs, and convenience of the bioassay procedure. The bioassay is widely applied to monitor a general toxicity of complex media, as well as a toxicity of individual chemical species [34,35,36,37,38] and radiation [40,41].

To evaluate molecular mechanisms of possible biological effects of the MNPs, enzymatic bioassays can be applied. The bioluminescence enzymatic assay is a relatively new direction in a toxicology practice [42,43,44]. The conventional enzymatic bioluminescent assay is based on the bacterial bioluminescent enzyme system, which involves two coupled enzymatic reactions:

(R1) $$NADH+FMN\overset{NADH:FMN-oxidoreductase}{\to }FMN\cdot H^{-}+NAD^{+}$$

(R2) $$FMN\cdot H^{-}+RCHO+O_{2}\overset{luciferase}{\to }FMN+RCOO^{-}+H_{2}O+hv$$

Similar to cellular bioassay, the enzymatic assays can assess a general toxicity in test samples. This toxicity type integrates all interactions of toxic compounds with the bioluminescent assay system: redox processes, polar and nonpolar binding, etc. Moreover, the enzymatic bioassay is specific to oxidizers due to its additional kinetic parameter, induction period, which depends on redox potentials of toxicants in solutions [44]. Therefore, the enzymatic bioassay can be additionally applied to monitor a toxicity of oxidative type, which is attributed to the redox properties of the toxic compounds only. Due to such features, the enzymatic bioassay can be used to reveal the ability of MNPs to interfere the redox reactions, as well as stimulate/inhibit biochemical processes as a result of surface hydrophobic/hydrophilic interactions.

Previously [45,46], we used the bioluminescent enzymatic assay system to evaluate the toxicities of general and oxidative types in solutions of organic and inorganic oxidizers (quinones and polyvalent metals, respectively). Changes in general toxicity and oxidative toxicity of aqueous media under exposure to humic substances were studied previously in [47,48,49]. Later [41,50,51,52], the toxicity and antioxidant activity of a series of fullerenols (i.e., carbon nanostructures, water-soluble derivatives of fullerenes, perspective pharmaceutical agents) were evaluated and compared. Effects of MNPs and MNPs with modified surface on bacterial enzyme reactions have not been studied yet.

Current paper compares the biological effects of MNPs with their zeta potentials and hydrodynamic diameters. Chemical precipitation method in situ and sol–gel synthesis were used to produce bare and modified MNPs. The MNPs were characterized by XRD, SEM, and DLS analysis. Toxicity effects of MNPs were evaluated and compared using a model cellular bioassay–luminous marine bacteria. Ability of MNPs to affect the biochemical processes was studied using a bioluminescence assay based on enzyme reactions of the luminous bacteria. A vital role of a ligand type (HA or APTES) in the effects of MNPs on the enzymatic activity was under consideration.

## 2. Materials and Methods

### 2.1. Preparations of Fe_3_O_4_ MNPs and Humic Acids- and Amino-Silica Functionalized Fe_3_O_4_ MNPs

#### 2.1.1. Preparation of the Fe_3_O_4_ MNPs

The bare Fe_3_O_4_ MNPs were prepared by the coprecipitation of Fe (II, III) salts in base solution as described in detail in [53]. Briefly, 7.56 g of FeCl_3_·6H_2_O (Sigma-Aldrich Chemie GmbH, Steinheim, Germany) and 2.78 g of FeCl_2_·4H_2_O (Sigma-Aldrich Chemie GmbH, Steinheim, Germany) were dissolved in 70 mL H_2_O, then added to 40 mL of 25% solution of ammonium hydroxide (Sigma-Aldrich Chemie GmbH, Steinheim, Germany) at 50 °C under argon flow with vigorous stirring at 600 rpm. The formed Fe_3_O_4_ nanoparticles were washed five times with Millipore water and ethanol to eliminate the impurities from synthesis followed by drying at 70 °C in vacuum.

#### 2.1.2. Preparation of the Silica-Coated Fe_3_O_4_/APTES Sample

To prepare the aminosilica-functionalized MNPs the surface modification of Fe_3_O_4_ was performed using 3-aminopropyltriethoxysilane (APTES, (Sigma-Aldrich Chemie GmbH, Steinheim, Germany)) as a silylation agent according to [54]. Briefly, 3.21 g of Fe_3_O_4_ was dispersed in 150 mL of ethanol/water (volume ratio, 1:1) solution. Then, 13.6 g of APTES was added into the solution under argon atmosphere, at 40 °C for 2 h; molar ratio of APTES to the Fe_3_O_4_ was applied as 4:1. After that, the solution was cooled down to a room temperature. The prepared Fe_3_O_4_/APTES MNPs were collected with a magnet (Nd, 0.3 T) and rinsed three times with ethanol and deionized water. Finally, the Fe_3_O_4_/APTES sample was dried in vacuum at 70 °C for 2 h.

#### 2.1.3. Preparation of the Humic Acids-Coated Fe_3_O_4_/HA Sample

The Fe_3_O_4_/HA sample was prepared by in situ method, which consists of the nucleation and growth of magnetite nanoparticles into humic acids matrix. The sample of HA was prepared from sodium humate (Powhumus (Humintech GmbH, Grevenbroich, Germany)). The low H/C atomic ratio (0.85) suggests high content of aromatic structures in the HA preparation. The total acidity of the sample was 5.3 mmol/g of acidic COOH and OH-groups, weight-average molecular weight (Mw) was 9.9 kD. The MNPs modified with HA (as Fe_3_O_4_/HA ratio 80:20 wt %) were prepared by the procedure described in [53,55].

### 2.2. Characterization of the MNPs

The phase composition and particle size of the MPNs samples were determined by X-ray diffraction analysis (XRD) in Bragg-Brentano geometry using a Philips X-Pert Diffractometer (Cr-Kα radiation, λ = 2.29106 Å (Philips Analytical, Eindhoven, The Netherlands)). The full width at a half maximum (FWHM) of the all reflections was used for particle size determination with the Scherrer equation. In order to quantify oxidation progress, the (440) reflection was fitted with five different functions in Origin 2019 Pro (OriginLab Corporation, Northampton, MA, USA). The morphology of the nanomaterials was investigated by scanning electron microscopy (SEM) using a Zeiss LEO SUPRA 25 microscope (ZEISS, Jena, Germany). The average particle diameter of the synthesized MNPs was determined by measuring the diameters of more than 100 particles with ImageJ software ± standard deviation. The data were fitted with Lognormal functions (95% confidence interval) using Origin 2019 Pro (Table S1, Supplementary Materials).

Dynamic light scattering (DLS) measurements were conducted with a NanoZS apparatus (Malvern Panalytical Ltd., Malvern, UK) at a wavelength of 633 nm with a solid-state He–Ne laser at a scattering angle of 173° at 25 °C. The intensity, volume, and number average diameters of MNP samples were calculated with the Zetasizer Nano 7.11 software (Brookhaven Instruments, Holtsville, NY, USA) utilizing an algorithm based on the Mie theory, which transforms time-varying intensities to particle diameters [56]. For the DLS analysis, each sample was diluted to approximately 0.1 g/L. Since the colloidal stability of the samples varies with the solution conditions, the particles may aggregate; all were measured at a given kinetic state achieved by 10 s of ultrasonication followed by 100 s of standstill. The average values of the hydrodynamic diameter were calculated from 3rd order cumulant fits of the correlation functions. The experiments were carried out in a disposable zeta cell (DTS 1070). The range of pH was between ~3 and ~10. The values of pH before and after the study were measured, subsequently; the value of pH after measurement by the method of dynamic light scattering was used. The experiments were performed at constant ionic strengths (0.01 M) set by NaCl. Data of zeta potential from pictures (dots) was fitted with Boltzmann functions (lines) and 95% confidence band in Origin 2019 Pro (Table S1, Supplementary Materials). Data of hydrodynamic size were fitted with Lognormal, Extreme, and Boltzmann functions (lines) for Fe_3_O_4_, Fe_3_O_4_/APTES, and Fe_3_O_4_/HA, respectively, and 95% confidence interval in Origin 2019 Pro (Table S1, Supplementary Materials).

### 2.3. Bioluminescence Assay Systems and Experimental Data Processing

Effects of the MNPs on microbiological and biochemical processes were evaluated using model bioluminescence assay systems, both cellular and enzymatic, i.e., luminous marine bacteria and a system of coupled enzymatic reactions of the marine bacteria, respectively.

The bacterial assay, i.e., Microbiosensor 677F, was based on the lyophilized luminous bacteria *Photobacterium phosphoreum* from the collection of the Institute of Biophysics SB RAS (CCIBSO 863), strain 667F IBSO. The enzyme preparation was based on the system of coupled enzyme reactions catalyzed by NADH:FMN-oxidoreductase from *Vibrio fischeri* (0.15 a.u.) and luciferase from *Photobacterium leiognathi* (0.5 mg/mL) [57]. The enzyme reactions (1) and (2) are presented in Introduction. All biological preparations were produced at the Institute of Biophysics SB RAS, Krasnoyarsk, Russia.

The following chemicals were used: NADH from MP Biomedicals, Santa Ana, CA, USA; FMN and tetradecanal from Sigma-Aldrich Chemie GmbH, St. Louis, MO, USA; 1,4-benzoquinone (Bq) from ACROS ORGANICS, NJ, USA; and sodium chloride (NaCl) from Khimreactivsnab, Barnaul, Russia. To construct the enzymatic assay system, we used 0.1 mg mL^−1^ enzyme preparation, 4 × 10^−4^ M NADH, 5.4 × 10^−4^ M FMN, and 0.0025% tetradecanal solutions. The enzymatic assay was performed in 0.05 M phosphate buffer, pH 6.8, at 20 °C.

Preparation of the MNPs suspensions for the bioluminescence analyses: Solid samples were ground in a mortar and dissolved in 3% aqueous NaCl solution or 0.05 M potassium phosphate buffer for bacterial or enzymatic assays, respectively. To obtain homogeneous suspensions, the 1000 mg/L stock suspensions were exposed to the ultrasound using an Elmasonic EASY 10 bath (Elma Schmidbauer GmbH, Singen, Baden-Wurttemberg, Germany) for 10 min. Analyzed MNPs concentrations (5–10^−13^ mg/L) were prepared from the stock suspensions, with additional 5 s ultrasonic treatment at every dissolution stage. Before the bioluminescence measurements, the dispersions were additionally treated in the ultrasonic bath for 5–10 s.

To evaluate the effect of optic filter on the MNPs’ suspensions, absorption spectra of the suspensions were recorded using UVIKON 943 Double Beam UV/VIS Spectrophotometer (Kontron Instruments, Milan, Italy). The suspensions of optical density higher than 0.1 (at 490 nm) were excluded, hence, the effect of “optic filter” [58] did not skew the results the bioluminescence measurements.

Measurements of bioluminescence intensity were carried out with bioluminometer Luminoskan Ascent (Thermo Electron Corporation, San Diego, CA, USA).

To assess the toxic/inhibitory effect of MNPs on the bacterial and enzymatic assays, the relative bioluminescence intensity, *I^rel^*, was calculated as:

(1) $$I^{rel}=I_{MNP}/I_{contr}$$

where *I_contr_* and *I_MNP_* are maximal bioluminescence intensities in the absence and presence of the MNPs, respectively. The experimental error for *I^rel^* did not exceed 8% in the bacterial assay and 12% in the enzymatic assay. The data for *I^rel^* processing were obtained with five samplings from all control and MNP solutions.

The dependence of *I^rel^* on MNP concentrations was studied. To characterize toxic effects of the MNPs on the bacteria and enzyme reactions, their effective concentrations that inhibited luminescence intensity by 20% (*I^rel^* = 0.8), *EC-20*, were determined and compared.

Additionally, the effects of the MNPs on the enzyme system were studied under conditions of an oxidative stress. An organic oxidizer, 1,4-benzoquinone (Bq), was chosen for these experiments to model conditions of the oxidative stress. The effective concentration of Bq that inhibited the bioluminescence intensity by 50% (8·10^−6^ M), was applied in all experiments.

**Figure 1:**
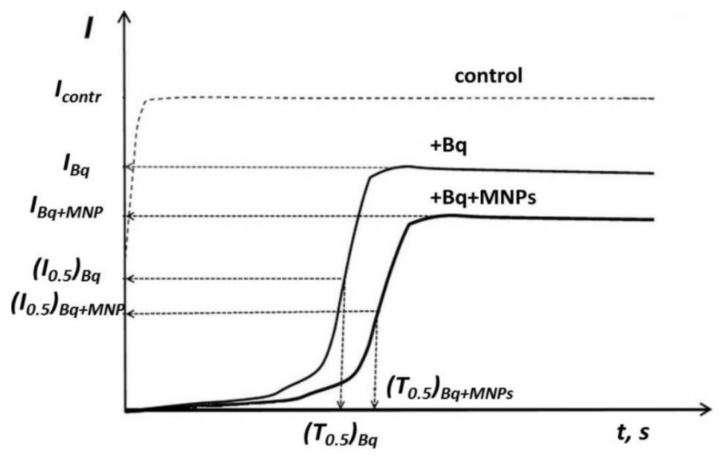
Bioluminescence kinetics in solutions of model oxidizer 1,4-benzoquinone (Bq) and magnetite nanoparticles (MNPs). Enzymatic assay.

To study and compare the effects of the MNPs under conditions of the oxidative stress, the relative bioluminescence intensity, *I^rel^_Bq_*, was calculated as:

(2) $$I_{Bq}^{rel}=I_{MNP+Bq}/I_{Bq}$$

where, *I_MNP+Bq_* and *I_Bq_* are maximal bioluminescence intensities in Bq solutions in the presence and absence of MNPs, respectively (see Figure 1). To characterize the MNP efficiency to suppress the enzymatic activity under the oxidative stress, the effective MNP concentrations that inhibited bioluminescence intensity by 50% (*I^rel^_Bq_* = 0.5), *EC-50*, were determined and compared.

Additionally, the specific effects of MNPs on redox processes in the enzymatic assay system under conditions of the oxidative stress were studied, i.e., the bioluminescence induction periods (*T*_0.5_)*_Bq_* in Bq solutions were measured (Figure 1). The *T^rel^_Bq_* values were calculated as:

(3) $$T_{Bq}^{rel}={\left(T_{0.5}\right)}_{Bq}/{\left(T_{0.5}\right)}_{Bq+MNP}$$

where (*T*_0.5_)*_Bq_* and (*T*_0.5_)*_Bq+MNP_* are bioluminescence induction periods in oxidizer (Bq) solutions in the absence and presence of MNPs, respectively (Figure 1).

The *I^rel^_Bq_* and *T^rel^_Bq_* values were plotted vs. concentrations of the nanoparticles.

Values of *I^rel^_Bq_* < 1 or *T^rel^_Bq_* < 1 revealed an increase in inhibition effects of the oxidizer solutions under the exposure to the MNPs. Values of *I^rel^_Bq_* ≈ 1 or *T^rel^_Bq_* ≈ 1 revealed the absence of the effect.

Experimental error for *I^rel^_Bq_* and *T^rel^_Bq_* did not exceed 12%. The data for *I^rel^_Bq_* and *T^rel^_Bq_* processing were obtained in two experiments with five samplings from all control and nanoparticle solutions.

Statistical processing of the biotesting results was carried out; *p*-values were calculated with RStudio using ANOVA and presented in Supplementary Materials, Tables S2–S5. The *p*-values were assessed by Student’s *t*-test of two independent sample distributions.

## 3. Results and Discussion

### 3.1. Microstructure of MNPs

**Figure 2:**
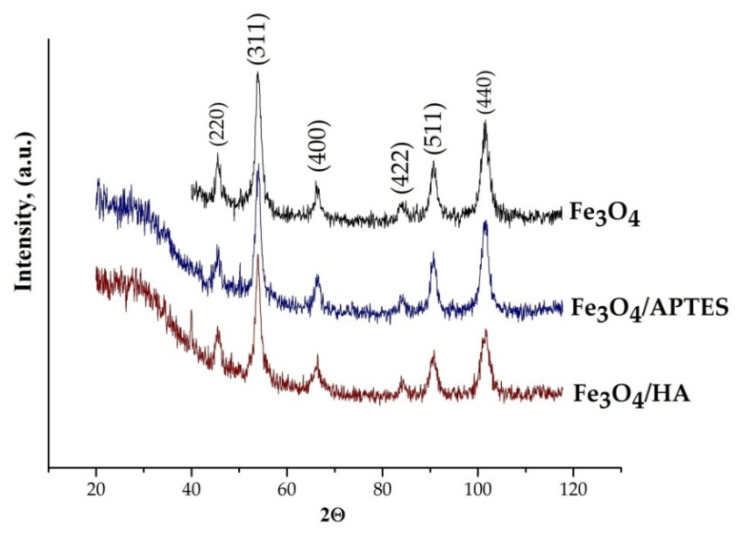
XRD patterns of MNPs samples.

The crystalline structures of the nanoparticles were identified with XRD analysis (Figure 2). The XRD patterns are similar for all samples and can be interpreted as a face-centered cubic (fcc) lattice with the parameters of 8.383(2), 8.372(1), and 8.382(6) Å for the Fe_3_O_4,_ Fe_3_O_4_/APTES, and Fe_3_O_4_/HA samples, respectively (Table 1).

The lattice parameters determined for all samples formulated in this study are smaller than those reported for magnetite 8.396–8.400 Å (ICDD–PDF 19–629), but larger than those for maghemite 8.33–8.34 Å (ICDD–PDF 39–1346). A plausible explanation of this phenomenon could be the process of partial oxidation of Fe^2+^ during drying and storage resulting in the nonstoichiometric Fe_3−δ_O_4_ formation where δ can range from zero (stoichiometric magnetite) to 1/3 (completely oxidized) [59]. For magnetite with an ideal Fe^2+^ content (assuming the Fe_3_O_4_ formula), the mineral phase is known as stoichiometric magnetite (x = 0.50). As magnetite becomes oxidized, the Fe^2+^/Fe^3+^ ratio (formula 4) decreases (x < 0.50), with this form denoted as nonstoichiometric or partially oxidized magnetite [59]. The stoichiometry can easily be converted to the following relationship:

(4) $$x=\frac{Fe^{2+}}{Fe^{3+}}=\frac{1-3\delta }{2+2\delta },$$

Finally, the composition of crystalline component of the samples can be assigned as follows: Fe_2,94_O_4_, Fe_2,88_O_4_, and Fe_2,93_O_4_ for the Fe_3_O_4_, Fe_3_O_4_/APTES, and Fe_3_O_4_/HA samples, respectively (Table S6, Supplementary Materials). A decrease in the magnetite content in the sample with a silica shell Fe_3_O_4_/APTES can be associated with the oxidation of magnetite in the synthesis process.

The coherent-scattering region size was derived from powder XRD data by Scherrer’s method. According to the formulas of Scherrer, Wolfe-Bragg, and formula for calculating the unit cell parameter, the value of the unit cell parameter a and the average particle size is strongly influenced by the angle at which the reflexes were detected (Q) and the full width at half maximum of XRD reflexes (FWHM). The original data is a set of points, for which it is rather difficult to determine Q and FWHM exactly. To calculate these parameters more accurately, the baseline points were described using five mathematical parameters using to R^2^ and χ^2^ as standard statistics parameters for fitting. The full width at half maximum of the reflections was used for particle size determination. The reflections were fitted with five models: Gauss function, Lorenz function, Voigt function, Pseudo-Voigt function, and Pearson VII function in Origin 2019 Pro (Figure S1, Supplementary Materials). According to R^2^ and χ^2^, data for Fe_3_O_4_ and Fe_3_O_4_/HA samples were most accurately fitted by the Pseudo-Voigt function (R^2^ = 0.9098 (9) and χ^2^ = 11.3893 (5), R^2^ = 0.9361 (2), and χ^2^ = 10.2240 (8), Table S7 in Supplementary Materials), Fe_3_O_4_/APTES—by the Pearson VII function (R^2^ = 0.9116 (4) and χ^2^ = 49.9908 (7), Table S7 in Supplementary Materials).

**Figure 3:**
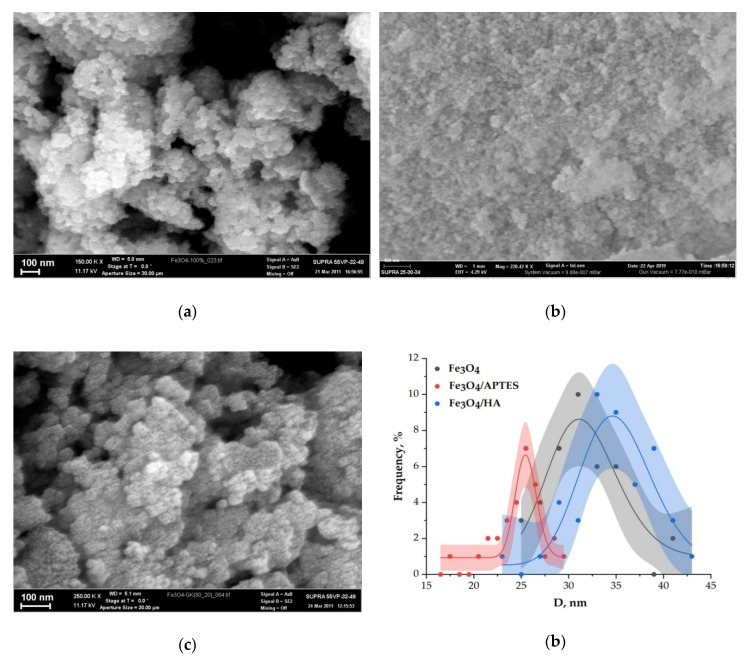
SEM images: (**a**) Fe_3_O_4_, (**b**) Fe_3_O_4_/APTES, and (**c**) Fe_3_O_4_/HA and (**d**) particle size distribution of samples determined from SEM analysis.

Changes in size of nanoparticles upon coating studied by SEM and XRD correlated. Although the spherical particle shape remained constant for all modification routes, a slight particle growth can be observed (Figure 3). For XRD, we used a spherical shape factor (0.94), and as a result, the smaller particle diameters were determined from SEM images. The bare magnetite particles exhibit a median particle diameter of 32.1 and 6.9 nm for SEM and XRD analysis, respectively. Such magnetite particles for the magnetic iron oxide nanoparticles are known to be for superparamagnetic MNPS with a high saturation magnetization and a high specific surface area [60,61].

The diameter of the Fe_3_O_4_/APTES particles changed to 9.6 and 24.18 nm compared to bare MNPs, whereas the Fe_3_O_4_/HA particles demonstrated a diameter gain to 10.3 and 34.75 nm for SEM and XRD, respectively. The particle growth can be explained by the phase distortion of the original inverse spinel, migration of iron ions to the surface, and subsequent oxidation [62]. Indeed, the XRD patterns confirm preservation of the spinel structure during both modification processes: content of magnetite decreases from ~80% to ~75% and ~60% for Fe_3_O_4_/HA and Fe_3_O_4_/APTES, respectively [63]. According to the coefficient of variation CV and standard deviation value σ of samples for SEM, Fe_3_O_4_/APTES has the smaller size distribution (11.6%, 2.8) than Fe_3_O_4_ (13.5%, 4.33) and Fe_3_O_4_/HA (12.45%, 4.33); all samples are considered to be polydisperse [64].

### 3.2. Characteristics of Surface and Hydrodynamic Size

Characteristics of surface charging and hydrodynamic size of MNPs define their biological activity. The interval of pH 6–7 is particularly important for our study as it provides proper conditions for bioassay procedures described in Section 3.3.

**Figure 4:**
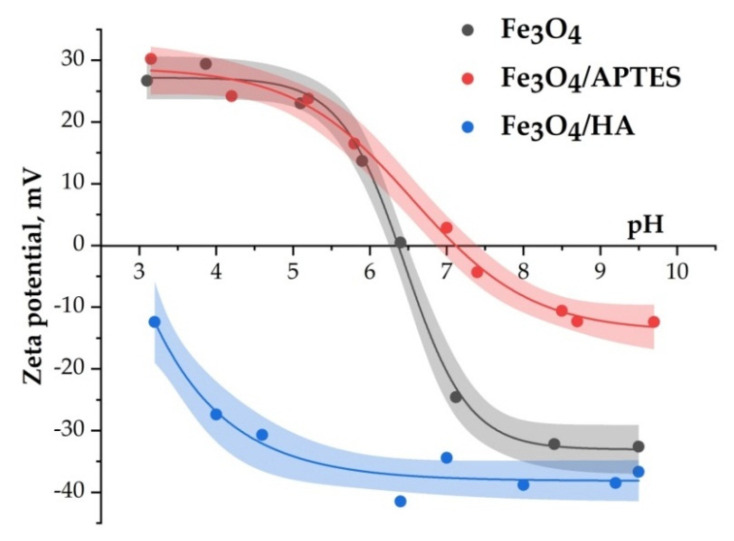
Zeta potential of Fe_3_O_4_, Fe_3_O_4_/APTES, and Fe_3_O_4_/HA nanoparticles as a function of pH (0.01 M KCl).

The particle charge measured as zeta potential is presented in Figure 4 for the Fe_3_O_4_, Fe_3_O_4_/APTES, and Fe_3_O_4_/HA nanoparticles. The charge of native magnetite reverses from positive to negative at pH ∼6.3, which may be considered as isoelectric point (IEP) in accordance with literature values [65]. The reactions of surface Fe–OH sites of magnetite, which can lead to formation of positive (Fe–OH_2_^+^) and negative (Fe–O^−^) surface charges, were observed earlier [5]. In the absence of any steric stabilizing layer, the naked Fe_3_O_4_, the uncoated sample is only prone to electrostatic stabilization. The zeta potential values were measured between +15 and −30 mV for pH 6–8, and the naked sample showed worse stability than the MNP samples coated with APTES and humic acids (Figure 4). The Fe_3_O_4_/APTES sample showed IEP value at pH 7.1, whereas the Fe_3_O_4_/HA sample showed IEP value at pH 3. According to [13], APTES has an IEP at pH 10.05. The variations in data for analogous samples in different studies probably indicate the dependence of the zeta potential value on the synthesis approach.

**Figure 5:**
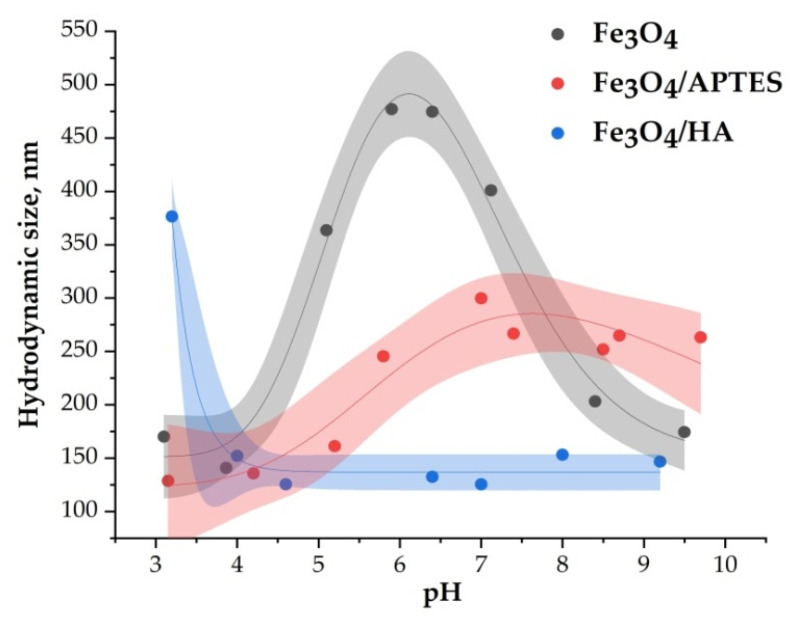
Average hydrodynamic diameter of MNPs nanoparticles as a function of pH (0.01 M KCl).

It is known that the presence of amino groups on the surface of Fe_3_O_4_ should shift the IEP towards higher pH values [12]. The shift of IEP value of Fe_3_O_4_/APTES to pH 7 (Figure 4) was attributed to the presence of protonated amino groups on the SiO_2_ surface [66]. Additionally, the position of zeta potential curve shows fewer negative charges in the alkaline region on the surface of Fe_3_O_4_/APTES, as compared to bare Fe_3_O_4_. The reactions of surface Fe_3_O_4_–O–Si–NH_2_ sites with H^+^ and OH^−^ ions lead to the formation of positive (Fe–O–Si–NH_3_^+^) surface charges. The zeta potential of the amino-functionalized MNPs sample via APTES varied drastically between +20 and −10 mV with pH in the given region. However, despite the low surface zeta potential as 0 to −10 mV at pH 7–8, aggregation of MNPs is negligible according to the magnitude of the hydrodynamic diameter varying from 250 to 300 nm (see in Figure 5). Presumably, the ethyl groups of APTES provide steric stabilization, generating an electrosterically stabilized system that confers proportionally larger stability as compared to the pure electrostatic stabilization by the protonated amino groups attached chemically to MNPs’ surface at low pH.

The humic acids-coated MNP_S_ are characterized by a negative zeta potential (between −35 and −40 mV) in the bioassay pH interval of 5.8–7.6 (Figure 4). For this pH range, the zeta potential value (between −35 and −40 mV) for humic acids-coated MNP_S_ shows the high degree of repulsion between the charged nanoparticles coating by anionic polyelectrolyte humic acids (pK_1_ = 4.8 and pK_2_ = 10, the total acidity of the sample is 5 mmol/g of acidic COOH and OH-groups with pK_1_ = 4.8 and pK_2_ = 10, respectively) in the dispersion. Thus, Fe_3_O_4_/HA MNPs with high zeta potential values provide the system’s stability in a way of combined steric and electrostatic effects integrated with steric hindrance due to the thick layer of macromolecular humic acids adsorbed on MNPs. A negative charge on the surface of Fe_3_O_4_/HA in the pH range of 3–10 indicates the complete coverage of the surface of magnetic nanoparticles with humic acids.

The average hydrodynamic diameter of MNPs depends on the value of pH. In the pH range of bioactivity testing (pH = 6–8), the samples of Fe_3_O_4_, Fe_3_O_4_/APTES, and Fe_3_O_4_/HA have an average hydrodynamic diameter of 100–500 nm (Figure 5). For bare Fe_3_O_4_, aggregation of nanoparticles becomes significant in this pH interval, due to the absence of electrostatic stabilization near IEP, where the surface charge density of particles is very low. The measured hydrodynamic diameter raises to 500 nm (Figure 5), even at low ionic strength. Modification of Fe_3_O_4_ by HA led to a significant decrease of hydrodynamic diameter to ~120 nm at pH 4–10.

### 3.3. Effects of MNPs on Bioluminescence of Cellular and Enzyme Assay Systems

We examined bioeffects of three types of MNPs, i.e., the bare magnetite nanoparticles (Fe_3_O_4_), and those modified by silica (Fe_3_O_4_/APTES) and humic acids (Fe_3_O_4_/HA). Cellular and enzymatic bioluminescent assay systems (Section 3.3.1 and Section 3.3.2, respectively) were used to study the bioeffects. Suppression of the bioluminescence is concerned with inhibition of membrane and intracellular processes in the bacterial cells or with inhibition of chemical and biochemical reactions in the enzyme system. The results might be considered as a model of MNP effects on microorganisms and their enzymatic processes in natural ecosystems. Additionally, we studied MNP effects under conditions of an oxidative stress, i.e., in the presence of a model oxidizer, Bq (Section 3.3.2.2). A role of ligand type (APTES or HA) in the catalytic activity of the bacterial enzymes was one of our main focuses.

#### 3.3.1. Effects of MNPs on Bacterial Cells

**Figure 6:**
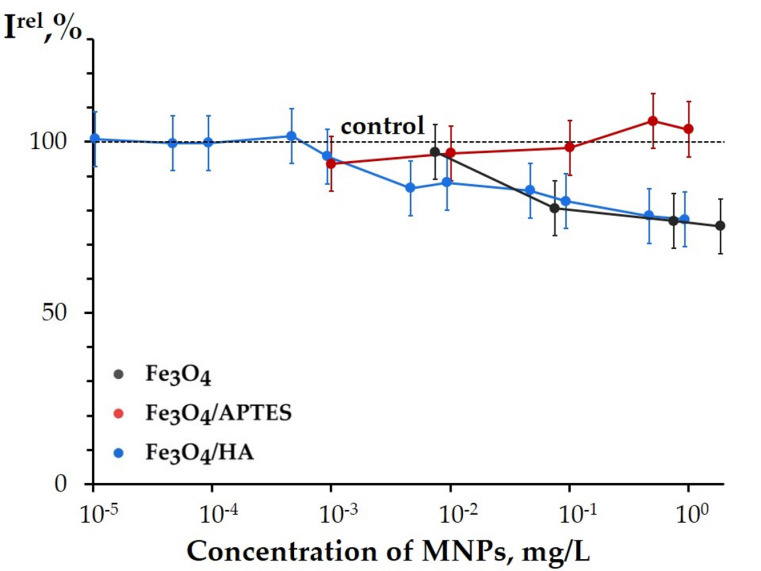
Relative bacterial bioluminescence intensity, ***I^rel^***, vs. concentration of MNPs.

Luminescence intensity of bacterial cells was studied in the presence of MNPs; concentrations of bare Fe_3_O_4_, Fe_3_O_4_/APTES, and Fe_3_O_4_/HA varied as shown in Figure 6. The effects for low MNP concentrations (<2 mg/L) were analyzed. The analysis of higher concentrations of MNPs was limited by their solubility in water and the dispersion stability during the time of the experiments. Additionally, we had to avoid the effect of “optic filter” which limits application of the luminescence signal registration in high optical density solutions and/or light scattering suspensions [58].

Figure 6 shows that no significant inhibition of the bacterial bioluminescence intensity was observed for the presented samples of MNPs; EC-50 values were not found experimentally. This indicates that MNPs did not result into a high toxic impact on bacterial cells in the concentration range applied. Therefore, the native limitation of MNP content in the water dispersions provides a low inhibitory effect on the bacterial luminescence, in contrast to fine solutions of compounds with higher solubility studied earlier, i.e., metals, their oxides, and organic chemicals [39,67]. The authors reported EC-50 values as 3.5–21 mg/L for soluble rare earth elements, ≤3 mg/L for dopant metals (Ni^2+^, Fe^3+^), and no toxicity for metal oxides (EC-50 > 500 mg/L).

Figure 6 shows that 20% decrease of the bacterial luminescence intensity was observed for bare Fe_3_O_4_ (*p* < 0.05 at C > 10^−2^ mg/L, Table S4). This indicates a moderate toxicity of bare Fe_3_O_4_ for the bacteria. This effect can be explained by a release of surface Fe^2+^ to media [68,69] with its following oxidation to Fe^3+^ in water solutions. The similar oxidative effect of Fe^3+^ on luminous bacteria was demonstrated earlier in [48,67].

The absence of inhibitory effect of silica-coated MNPs (Fe_3_O_4_ /APTES) is evident from Figure 6 (*p* > 0.05). This result might be related to the specific characteristics of the silica material used for modification of Fe_3_O_4_ surface: higher hydrophobicity and low redox activity (due to low surface charge with zeta potential interval of +15 to −10 mV). Probably, the silica cover can neutralize the toxic effect of Fe_3_O_4_ surface by preventing it from dissolution of surface iron due to covalent binding. A confirmation of this hypothesis was presented previously in [69]: Swindle et al. showed that organic coatings of the MNP surface protects the particles from the dissolution.

Humic acids-coated MNPs, Fe_3_O_4_/HA, demonstrated 20% inhibition of the bioluminescence intensity (*p* < 0.05 at C > 10^−3^ mg/L), similar to bare Fe_3_O_4_, Figure 6. Hence, modification of magnetite surface with HA did not protect the bacteria from the toxic effect, in contrast to the silica-modified magnetite. The peculiarity of its effect is a wide concentration interval of 20% inhibition (ca. 5 × 10^−3^–1 mg/L).

Bioeffects of humic substances were observed earlier [47,70]. In [48,49], the inherent inhibitory effect of humic substances on luminous bacteria was found at concentrations higher than 10 mg/L. This value shows that the toxic effect of humic acids-coated MNPs on bacterial cells is not defined by the HA, but by the composite of Fe_3_O_4_/HA.

A membrane activity of Fe_3_O_4_/HA probably can be responsible for its toxic effect on the bacteria: the hydrodynamic diameter of Fe_3_O_4_/HA (100 nm) is smaller than the other systems’ diameters (300 nm for Fe_3_O_4_/APTES and 500 nm for the bare Fe_3_O_4_) providing a higher penetrating ability for the humic acids-coated nanoparticles. Unlike the other systems, the surface charge of the Fe_3_O_4_/HA sample is constantly high and negative (−35 to 40 mV) in the biotesting pH range of 6–7 (Figure 4). However, remarkable variations in zeta potential are not observed likely due to the lower sensitivity of the electrophoretic light scattering method in comparison with the cellular sensitivity. Sensitive Mossbauer spectroscopy showed minor changes in composition of solid Fe_3_O_4_-HA due to iron ions release [71,72].

As an outline, a moderate toxicity of bare Fe_3_O_4_ was found at concentrations < 2 mg/L; it was supposedly attributed to the oxidative effect of the surface and dissolved Fe^3+^ in the solutions. High negative surface charge, lower size, and membrane activity, as well as iron ions release, are potential reasons for the moderate effect of Fe_3_O_4_/HA. The toxic effect of silica-coated Fe_3_O_4_ (Fe_3_O_4_/APTES) was absent, thus revealing an inertia of the hydrophobic coating.

#### 3.3.2. Effects of the MNPs on Enzyme Reactions of Luminous Bacteria

##### 3.3.2.1. Effects of the MNPs on the Bioluminescence System of Coupled Reactions

**Figure 7:**
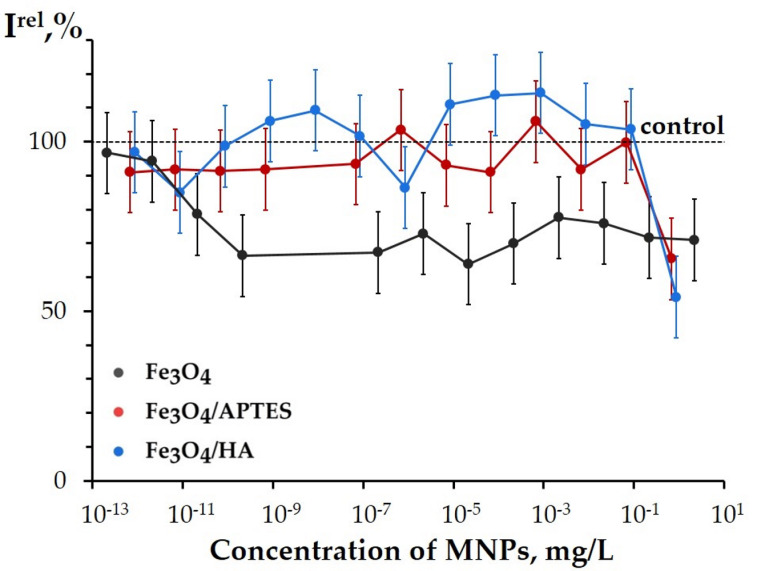
Relative enzyme bioluminescence intensity, ***I^rel^***, vs. concentration of MNPs.

Similar to luminous marine bacteria (Section 3.3.1), the luminescence intensity of the bacterial enzymatic system was studied in the presence of Fe_3_O_4_, Fe_3_O_4_/HA, and Fe_3_O_4_/APTES; low nanoparticle’ concentrations (10^−13^–2 mg/L) were applied (Figure 7). The effective concentrations EC-20 of Fe_3_O_4_/HA and Fe_3_O_4_/APTES appeared to be comparable—ca. 1 mg/L, whereas the 20% inhibition of the enzymatic bioluminescence by bare Fe_3_O_4_ was found in a wide concentration range: 10^−11^–2 mg/L.

Inhibition of the enzyme bioluminescence system by Fe_3_O_4_ was statistically reliable (*p* < 0.05, Table S2), and the inhibitory effect was not concentration dependent in the used range (*p* < 0.05; Figure 7). Probably, interactions of the enzymes with the surface of Fe_3_O_4_ promote the inhibition of the enzymatic reactions. Additionally, a release of iron ions to water media, discussed before [68,69], can contribute to the specific inhibition of the bioluminescence intensity. Earlier [46,48], the inhibition of the bioluminescence enzymatic system by Fe^3+^ was demonstrated.

At low concentrations (<10^−1^ mg/L), no inhibition effects were found in the solutions of modified MNPs: Fe_3_O_4_/APTES and Fe_3_O_4_/HA (*p* > 0.05). Hence, coating the MNP surface with polyelectrolytes APTES and HA prevents from the inhibition of the enzyme reactions, probably, by excluding the enzyme interactions with iron atoms of the MNP surface.

It is evident from the data presented that inhibitory ability of Fe_3_O_4_/HA differed in cellular and enzymatic assay systems: humic acids coating protected from the enzyme inhibition, but does not protect from the cell suppression (compare Figure 6 and Figure 7). This comparison likely supports a suggestion on the membrane-active mechanism of toxicity of Fe_3_O_4_/HA for bacterial cells. On the other hand, the difference in results could be additionally explained with the acidity regimes in the assay solutions: the enzymatic assay was conducted in a phosphate buffer (pH 6.8), while the bacterial assay was carried out in NaCl solution. The pH regimes might be critical for the toxicity of HA-coated MNPs suspension, and they should be further studied.

##### 3.3.2.2. Effects of MNPs on the Bioluminescence System of Coupled Reactions under Conditions of Oxidative Stress

Since the conditions of water purification with MNPs might vary, the biological activity of MNP surface under the oxidative stress is of a special interest.

The oxidative stress was chemically modeled in the bioluminescence enzymatic assay. To provide this, the bioluminescence kinetics of the enzyme system was studied in solutions of a model organic oxidizer (1,4-benzoquinone, Bq, 8·10^−6^ M) and MNPs. Concentration of MNPs varied at a low concentration range of 10^−13^–5 mg/L; the effect of “optic filter” was excluded.

**Figure 8:**
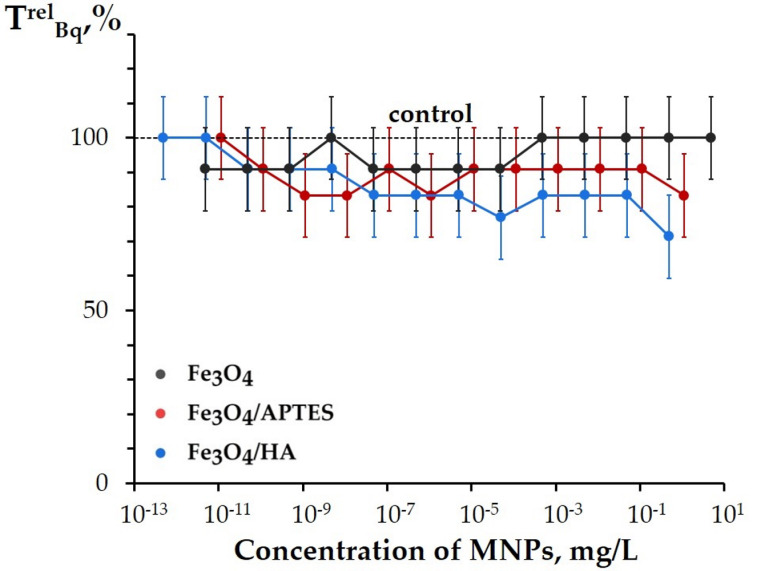
Relative bioluminescence induction period in 1,4-benzoquinone solution (8·10^−6^ M), *T^rel^_Bq_*, vs. concentration of MNPs.

**Figure 9:**
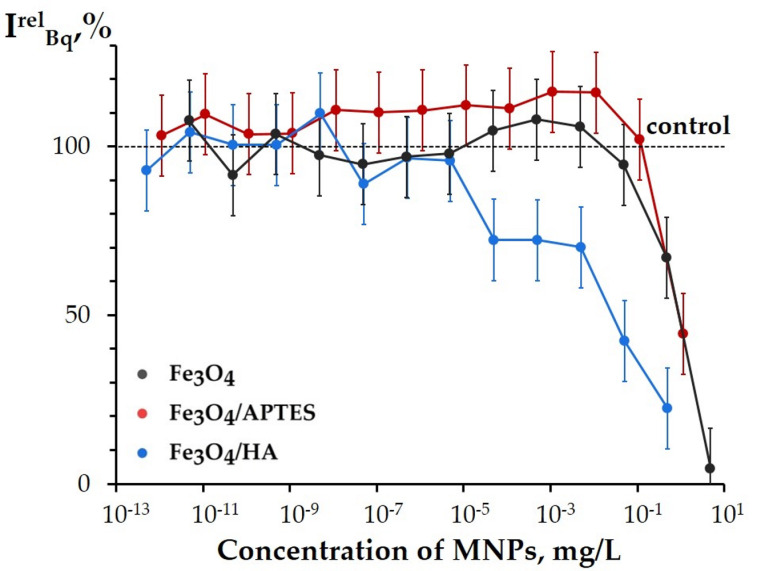
Relative bioluminescence intensity of enzyme system in 1,4-benzoquinone solution (8·10^−6^ M), *I^rel^_Bq_*, vs. concentration of MNPs.

The bioluminescence intensity and the induction period were monitored (see Figure 1 in Section 2.3). The relative bioluminescence intensity, *I^rel^_Bq_*, and relative induction period, *T^rel^_Bq_*, were calculated at different concentrations of MNPs and presented in Figure 8 and Figure 9.

Figure 8 demonstrates the *T^rel^_Bq_* values of MNPs. Bare Fe_3_O_4_ and Fe_3_O_4_/APTES did not show the reliable deviations from the control samples (*p* > 0.05; Table S5; Figure 8). This result reveals an independence of the bioluminescence induction period (*T*_0.5_, Figure 1) on the MNP concentrations and demonstrates, by this, that the MNPs do not noticeably change the enzymatic redox activity under the conditions of the oxidative stress.

Only humic acids-coated MNPs, Fe_3_O_4_/HA (Figure 8), demonstrated low, but reliable deviations from the control samples (*T^rel^_Bq_* < 1) (*p* < 0.05 at C > 5 × 10^−9^ mg/L). This effect was constant in a wide range of low concentrations, similar to this presented earlier in Figure 6 and Figure 7. Hence, the results demonstrate that Fe_3_O_4_/HA additionally increased the oxidative feature of the Bq solutions. This effect might be explained with a partial hydrolyzing of HA coating in the Bq solutions and, as a result, higher content of oxidized iron (Fe^3+^, surface and/or free) in the suspensions of Fe_3_O_4_/HA, as compared to bare Fe_3_O_4_. Peculiarities of these processes are previously discussed in [71,72,73,74,75,76,77,78], as summarized below:

Dissolution of the solid phase and an increase in dissolved Fe^2+^ and/or Fe^3+^ as a result of incubation of MNPs with humic substances was demonstrated by Sundman et al. in [71]. Authors revealed that the magnetite incubated with native humic substances becomes more oxidized as compared with a control magnetite [71]. The reason for iron ions release from Fe_3_O_4_/HA matrix is the supramolecular nature of HA that means associations via weak hydrophobic (van der Waals, π-π, CH-π) and hydrogen bonds [73]. As a result of different treatments (including an oxidative stress), HA can be hydrolyzed, thus leading to the destruction of the protective layer of Fe_3_O_4_/HA. Derivatives of HA contribute into the iron ions release from magnetite. Both iron ions, Fe^2+^ and Fe^3+^, form strong mixed ligand complexes with HA [72,74] and/or enzymes [75]. Therefore, thermodynamically driven dissolution and subsequent complexation reactions between HA, enzymes, and iron ions can be an important reason for Fe_3_O_4_/HA dissolution. The dissolution of Fe_3_O_4_/HA was also supported by hydrodynamic particle size analysis via DLS showing an increase in the particle size [76,77].

The studies of the Fe_3_O_4_/HA bioeffects provides an understanding of a role of humic substances in the biogeochemical cycles of iron (Fe^2+^/Fe^3+^). These are naturally important processes, since humic substances are main components of dissolved organic matter and, therefore, play an important role in a complex formation of colloidal magnetite particles, redox activity of iron in these particles, and their effect on microbiota. In nature, humic substances are known to be sorbed onto MNPs, change MNP’ surface charges, affect the aggregation and bioavailability [79], interfere with mineral dissolution/precipitation reactions [80], and drive redox reactions [81].

In contrast to Fe_3_O_4_/HA, water solutions of HA did not show any low-concentration inhibitory effects under the conditions of the oxidative stress, but opposite, revealed an antioxidant activity and mitigation of the oxidative effect of Bq (i.e., *T^rel^_Bq_* > 1) [49,52,58]. Hence, humic acids-coated MNPs, Fe_3_O_4_/HA, show higher oxidative toxicity than its components Fe_3_O_4_ or HA under the conditions of the oxidative stress.

Figure 9 presents the *I^rel^_Bq_* values at different concentrations of the MNPs. This parameter (Equation (2)) analyses the bioluminescence intensity, *I*, of the enzymatic system (Figure 1 in Section 3.2); it depends on the combination of physicochemical mechanisms (redox, polar, and apolar interactions) under conditions of the model oxidative stress.

The *I^rel^_Bq_* values demonstrated the inhibition of the bioluminescence intensity at higher concentrations of all MNPs (*p* < 0.05; concentration intervals for statistical confidence are presented in Table S3; Figure 9). All MNPs did not show a reliable activation of the bioluminescence enzyme system (i.e., no mitigation of the oxidative stress) at low concentrations; even Fe_3_O_4_/APTES showed the *p* > 0.05 at 10^−7^–10^−1^ mg/L.

No statistical difference was found between the effects of bare Fe_3_O_4_ and Fe_3_O_4_/APTES under the conditions of the oxidative stress (*p* > 0.05).

The parameters of *I^rel^_Bq_*, Figure 9, allowed us to determine the effective concentrations of EC-50 using the experimental data, in contrast to the bioluminescence parameters analyzed before (*I^rel^* in the bacterial assay, *I^rel^* in the enzymatic assay, and *T^rel^_Bq_* in Figure 5, Figure 7, and Figure 8, respectively). So, *I^rel^_Bq_* appeared to be most sensitive bioluminescence parameter to monitor the effects of MNPs. The EC-50 were determined as 1, 1, and 5 × 10^−2^ mg/L for the Fe_3_O_4_, Fe_3_O_4_/APTES, and Fe_3_O_4_/HA, respectively. It is seen that humic acids-coated MNPs, Fe_3_O_4_/HA, showed maximal inhibitory activity under the oxidative stress. Additionally, in a wide interval of low concentrations (5 × 10^−6^–5 × 10^−1^ mg/L), the Fe_3_O_4_/HA demonstrated higher ability to inhibit the bioluminescence intensity (Figure 9), similar to the other kinetic bioluminescence parameter of the enzyme system, *T^rel^_Bq_* (Figure 8), discussed before.

Comparison of Figure 8 and Figure 9 reveals that the parameter *I^rel^_Bq_* is more sensitive to MNPs than *T^rel^_Bq_*. Since the *T^rel^_Bq_* is attributed to redox characteristics of solutions only [44,45,82], the sensitivity of the *I^rel^_Bq_* parameter might be explained by contribution of hydrophobic interactions to the enzymatic activity under the conditions of oxidative stress.

As an outline for Section 3.3.2.2: under the conditions of the model oxidative stress (i.e., in the presence of the model organic oxidizer, Bq), all MNPs, bare and modified, did not reveal an antioxidant activity. Moreover, humic acids-coated MNPs demonstrated the additional inhibitory effect on the enzyme reactions in a wide concentration range (Figure 8 and Figure 9), which can be explained with HA hydroxylation, a destruction of the protective layer, and a higher content of iron Fe^3+^ in the samples of Fe_3_O_4_/HA, as compared to bare Fe_3_O_4_ [78].

## 4. Conclusions

We studied effects of MNPs on luminous marine bacterium and its enzymatic reactions. Three types of MNPs with different surface characteristics were under investigation: bare Fe_3_O_4_, as well as Fe_3_O_4_ modified with 3-aminopropyltriethoxysilane (Fe_3_O_4_/APTES) and humic acids (Fe_3_O_4_/HA). Inhibition effects of the MNPs were studied and compared in a low-concentration range (<2 mg/L).

Bacterial bioassay showed a moderate toxicity of bare Fe_3_O_4_ and Fe_3_O_4_/HA. Based on literature data, we hypothesized that the toxicity of bare Fe_3_O_4_ is attributed to the oxidative effect of the surface and free Fe^3^^+^ in the suspensions. Toxicity of Fe_3_O_4_/HA was supposedly concerned with a high negative surface charge, lower size, and membrane activity of the nanoparticles. The enzymatic bioassay revealed inhibitory ability of bare Fe_3_O_4_ only.

Additionally, the enzymatic assay was applied to study inhibitory ability of the nanoparticles under conditions of a model oxidative stress (i.e., in the solutions of model oxidizer, 1,4-benzoquinone). We demonstrated that the oxidative stress increases a sensitivity of the enzymatic assay system to MNPs: the bioluminescence intensity determined under the conditions of an oxidative stress, *I^rel^_Bq_*, was found to be most sensitive parameter to monitor the inhibitory effects of MNPs. Humic acids-coated MNPs (Fe_3_O_4_/HA) showed maximal inhibitory effect on the enzyme reactions, probably due to the partial dissociation of HA coating in the oxidant’ solutions and, as a result, higher content of oxidized iron Fe^3+^ in the suspensions.

Silica surface modification made the magnetite nanoparticles (Fe_3_O_4_/APTES) inert for both bacteria and their enzymes (Figure 6 and Figure 7). Since silica in free and combined forms is a dominant component of many solid soils and soil solutions/dispersions, our results elucidate the biological function of silica in the biogeochemical cycling of iron.

Our results show that the bioluminescence assays, cellular and enzymatic, can serve as promising and highly convenient tools to evaluate and compare the bioavailability of Fe^3+^ ions in natural dispersions of iron-containing nanoparticles—magnetite, ferrihydrite, so on. The following studies should reveal the quantitative dependences of the bioluminescence response vs. a release of Fe^3+^ from these MNPs. We have already previously detected the significant iron dissolution in the presence of Fe_3_O_4_ coated by other ligands [83].

## Figures and Tables

**Table 1:** Microstructure of magnetite nanoparticles (MNPs).

Sample	Fe_3_O_4_	Fe_3_O_4_/APTES	Fe_3_O_4_/HA
**Structure**	Fe_2,94_O_4_	Fe_2,88_O_4_	Fe_2,93_O_4_
**D_XRD_, nm ^1^**	6.9 ± 2.4	9.6 ± 1.4	10.3 ± 1.3
**CV, % ^2^**	34	14.5	12.6
**D_SEM_, nm ^3^**	32.1 ± 4.3	24.18 ± 2.8	34.75 ± 4.3
**CV, %**	13.5	11.6	12.45

^1,3^ D _XRD_ and D _SEM_—average particle size calculated by the Scherrer equation and SEM, respectively, ± standard deviation, nm. ^2^ CV—coefficient of variation characterizing the polydispersity of the system, %.

## Supplementary Materials

The following are available online at https://www.mdpi.com/2079-4991/10/8/1499/s1, Figure S1: fitting experimental data by different mathematical models; Table S1: statistic data of fitting by different mathematical models for SEM, zeta potential, and hydrodynamic size data; Table S2: statistics of relative enzyme bioluminescence intensity (Figure 7); Table S3: statistics of relative bioluminescence intensity of enzyme system in 1,4-benzoquinone solution (Figure 9); Table S4: statistics of relative bacterial bioluminescence intensity (Figure 6); Table S5: statistics of relative bioluminescence induction period in 1,4-benzoquinone solution (Figure 8); Table S6: microstructure of magnetite nanoparticles (MNPs); Table S7: statistical data of fitting by different mathematical models.

## Abbreviations

APTES	3-aminopropyltriethoxysilane
Bq	1,4-benzoquinone
CV	Coefficient of variation
DLS	Dynamic light scattering
ELS	Electrophoretic light scattering
FMN	Flavin mononucleotide
FWHM	Full width at a half maximum
HA	Humic acids
IEP	Isoelectric point
MNPs	Magnetite nanoparticles
NADH	Nicotinamide adenine dinucleotide disodium salt-reduced
SEM	Scanning electron microscopy
XRD	X-ray diffraction analysis
