# Supplementary material for: Effects of Modified Magnetite Nanoparticles on Bacterial Cells and Enzyme Reactions
Source: Nanomaterials (Basel). 2020 Jul 30;10(8):1499. doi: 10.3390/nano10081499 (PMC7466415; doi:10.3390/nano10081499)
Supplement: Supplementary file 1 [file nanomaterials-10-01499-s001.zip › Supplementary files/Table S1.pdf]

Table S1. Statistic data of fitting by different mathematical models for SEM, zeta potential and hydrodynamic size data

**Sample**	**SEM**		**Zeta potential**		**Hydrodynamic size**					
	**Lognormal functions**		**Boltzmann functions**		**Lognormal functions**		**Extreme functions**		**Boltzmann functions**	
	R^2	χ^2	R^2	χ^2	R^2	χ^2	R^2	χ^2	R^2	χ^2
**Fe_3O_4**	0.76	2.19	0.99	3.83	0.98	398.5	-	-	-	-
**Fe_3O_4/APTES**	0.80	0.86	0.99	3.52	-	-	0.85	607.9	-	-
**Fe_3O_4/HA**	0.73	3.16	0.92	6.80	-	-	-	-	0.97	172.5
