# Supplementary material for: Effects of Modified Magnetite Nanoparticles on Bacterial Cells and Enzyme Reactions
Source: Nanomaterials (Basel). 2020 Jul 30;10(8):1499. doi: 10.3390/nano10081499 (PMC7466415; doi:10.3390/nano10081499)
Supplement: Supplementary file 1 [file nanomaterials-10-01499-s001.zip › Supplementary files/Table S7.pdf]

Table S7. Statistic data of fitting by different mathematical models

			Fe_3O_4		
	**Lorenz function**	**Gauss function**	**Voigt function**	**Pseudo-Voigt function**	**PearsonVII function**
R^2	0.90868	0.90211	0.90956	0.90989	0.90965
χ^2	11.54172	12.37203	11.43064	11.38935	11.41964
			**Fe_3O_4/APTES**		
R^2	0.90834	0.91022	0.91136	0.91105	0.9116
χ^2	51.12376	50.77037	50.12376	50.3012	49.99087
			**Fe_3O_4/HA**		
R^2	0.93461	0.93082	0.93592	0.93612	0.93606
χ^2	10.46463	11.07169	10.25538	10.22408	10.2392
