# Supplementary material for: Effects of Modified Magnetite Nanoparticles on Bacterial Cells and Enzyme Reactions
Source: Nanomaterials (Basel). 2020 Jul 30;10(8):1499. doi: 10.3390/nano10081499 (PMC7466415; doi:10.3390/nano10081499)
Supplement: Supplementary file 1 [file nanomaterials-10-01499-s001.zip › Supplementary files/Table S3.pdf]

Table S3. Statistics of relative bioluminescence intensity of enzyme system in 1,4-benzoquinone solution (Figure 9)

**Samples**	**p**	
	**Concentrations**	
Fe_3O_4	0.0001	
Fe_3O_4/APTES	0.0001	
Fe_3O_4/HA	0.001	
	**Comparison by sample pairs**	
	**Concentration≤5E-06**	**Concentration>5E-06**
Fe_3O_4/APTES- Fe_3O_4	0.01	>0.05
Fe_3O_4/HA- Fe_3O_4	>0.05	0.001
Fe_3O_4/HA- Fe_3O_4/APTES	0.01	0.001

Bioactivity in relation to control:

**Samples**	**Concentraions, mg/L**												
Fe_3O_4-control	4.7E-12	4.7E-11	4.7E-10	4.7E-9	4.7E-8	4.7E-7	4.7E-6	4.7E-5	4.7E-4	4.7E-3	4.7E-2	4.7E-1	4.7E+0
	>0.05	>0.05	>0.05	>0.05	>0.05	>0.05	>0.05	>0.05	>0.05	>0.05	>0.05	0.0001	0.0001
	>0.05												
Fe_3O_4/APTES-control	1.1E-12	1.1E-11	1.1E-10	1.1E-9	1.1E-8	1.1E-7	1.1E-6	1.1E-5	1.1E-4	1.1E-3	1.1E-2	1.1E-1	1.1E+0
	>0.05	>0.05	>0.05	>0.05	>0.05	>0.05	>0.05	>0.05	>0.05	0.01	0.01	>0.05	0.0001
	>0.05												
Fe_3O_4/HA-control	4.9E-13	4.9E-12	4.9E-11	4.9E-10	4.9E-9	4.9E-8	4.9E-7	4.9E-6	4.9E-5	4.9E-3	4.9E-2	4.9E-1	
	>0.05	>0.05	>0.05	>0.05	0.01	>0.05	>0.05	0.01	0.01	0.001	0.0001	0.0001	
	0.01												
