# Supplementary material for: Effects of Modified Magnetite Nanoparticles on Bacterial Cells and Enzyme Reactions
Source: Nanomaterials (Basel). 2020 Jul 30;10(8):1499. doi: 10.3390/nano10081499 (PMC7466415; doi:10.3390/nano10081499)
Supplement: Supplementary file 1 [file nanomaterials-10-01499-s001.zip › Supplementary files/Table S4.pdf]

Table S4. Statistics of relative bacterial bioluminescence intensity (Figure 6)

Samples	p
	**Concentrations**
Fe_3O_4	>0.05
Fe_3O_4/APTES	>0.05
Fe_3O_4/HA	0.01
	**Comparison by sample pairs**
Fe_3O_4/APTES- Fe_3O_4	0.01
Fe_3O_4/HA- Fe_3O_4	>0.05
Fe_3O_4/HA- Fe_3O_4/APTES	0.01

Bioactivity in relation to control:

Samples	Concentrations, mg/L										
Fe_3O_4-control	7.5E-3	7.5E-2	7.5E-1	1.9E+0							
	>0.05	0.01	0.01	0.0001							
	0.01										
Fe_3O_4/APTES-control	1E-3	1E-2	1E-1	5E-1	1E+0						
	>0.05	>0.05	>0.05	>0.05	>0.05						
	>0.05										
Fe_3O_4/HA-control	1E-5	4.6E-5	9.3E-5	4.6E-4	9.3E-4	4.6E-3	9.3E-3	4.6E-2	9.3E-2	4E-1	9E-1
	>0.05	>0.05	>0.05	>0.05	0.01	0.01	0.01	0.001	0.001	0.001	0.001
	0.001										
