# Supplementary material for: Effects of Modified Magnetite Nanoparticles on Bacterial Cells and Enzyme Reactions
Source: Nanomaterials (Basel). 2020 Jul 30;10(8):1499. doi: 10.3390/nano10081499 (PMC7466415; doi:10.3390/nano10081499)
Supplement: Supplementary file 1 [file nanomaterials-10-01499-s001.zip › Supplementary files/Table S2.pdf]

Table S2. Statistics of relative enzyme bioluminescence intensity (Figure 7)

Samples	p
	**Concentrations**
Fe_3O_4	>0.05
Fe_3O_4/APTES	0.001
Fe_3O_4/HA	0.0001
	**Comparison by sample pairs**
Fe_3O_4/APTES- Fe_3O_4	0.001
Fe_3O_4/HA- Fe_3O_4	0.0001
Fe_3O_4/HA- Fe_3O_4/APTES	>0.05

Bioactivity in relation to control:

Samples	Concentrations, mg/L													
Fe_3O_4-control	2.1E-13	2.1E-12	2.1E-11	2.1E-10	2.1E-7	2.1E-6	2.1E-5	2.1E-4	2.1E-3	2.1E-2	2.1E-1	2.1E+0		
	0.001	0.001	0.0001	0.0001	0.0001	0.0001	0.0001	0.0001	0.0001	0.0001	0.0001	0.0001		
	0.0001													
Fe_3O_4/APTES-control	6.8E-14	6.8E-13	6.8E-12	6.8E-11	6.8E-10	6.8E-8	6.8E-7	6.8E-6	6.8E-5	6.8E-4	6.8E-3	6.8E-2	6.8E-1	
	>0.05	0.0001	0.0001	0.0001	0.0001	0.001	>0.05	0.0001	0.0001	>0.05	0.0001	0.001	0.0001	
	0.01													
Fe_3O_4/HA-control	8.5E-14	8.5E-13	8.5E-12	8.5E-11	8.5E-10	8.5E-9	8.5E-8	8.5E-7	8.5E-6	8.5E-5	8.5E-4	8.5E-3	8.5E-2	8.5E-1
	>0.05	>0.05	0.01	>0.05	>0.05	>0.05	>0.05	0.01	0.01	0.01	0.01	>0.05	>0.05	0.001
	>0.05													
