# Supplementary material for: Effects of Modified Magnetite Nanoparticles on Bacterial Cells and Enzyme Reactions
Source: Nanomaterials (Basel). 2020 Jul 30;10(8):1499. doi: 10.3390/nano10081499 (PMC7466415; doi:10.3390/nano10081499)
Supplement: Supplementary file 1 [file nanomaterials-10-01499-s001.zip › Supplementary files/Table S6.pdf]

Table S6. Microstructure of MNPs

Sample	Fe_3O_4			Fe_3O_4/APTES			Fe_3O_4/HA		
**hkl** [1]	**2Q, °** [2]	**d, Å** [3]	**FWHM, °** [4]	**2Q, °**	**d, Å**	**FWHM, °**	**2Q, °**	**d, Å**	**FWHM, °**
**220**	45.60	2.971	3.877(5)	45.61	2.959	1.790(1)	45.67	2.966	1.591(2)
**311**	53.98	2.535	1.445(2)	54.01	2.525	1.870(1)	54.01	2.535	1.344(7)
**400**	66.28	2.094	2.558(2)	66.41	2.095	2.980(2)	66.36	2.094	1.401(2)
**422**	84.25	1.714	5.912(6)	84.37	1.711	1.870(5)	84.25	1.714	1.483(2)
**511**	90.77	1.610	1.541(2)	90.68	1.609	2.749(5)	90.71	1.612	1.792(3)
**440**	101.52	1.476	2.248(7)	101.6	1.479	0.860(4)	101.4	1.476	1.591(2)
**a**, Å [5]	8.383(2)			8.372(1)			8.382(6)		
**X** [6]	0.387(7)			0.290(3)			0.382(1)		
**δ** [7]	0.059(4)			0.117(2)			0.062(7)		
**Structure**	$Fe_{2,94}O_4$			$Fe_{2,88}O_4$			$Fe_{2,93}O_4$		
**D_{XRD}, nm** [8]	6.9±2.4			9.6±1.4			10.3±1.3		
**CV, %** [9]	34			14.5			12.6		
**D_{SEM}, nm** [10]	32.1±4.3			24.18±2.8			34.75±4.3		
**CV, %**	13.5			11.6			12.45		

[1] hkl – Miller indexes.
[2] Q – angle at which the reflex was measured.
[3] d – interplanar distance.
[4] FWHM – full width at half maximum of XRD reflex.
[5] a – interplanar distance.
[6] X – the Fe^{2+}/Fe^{3+} ratio.
[7] δ – calculated value, which range from zero (stoichiometric magnetite) to 1/3 (completely oxidized).
[8] D_{XRD} – average particle size calculated by the Scherrer equation ± standard deviation.
[9] CV – coefficient of variation characterizing the polydispersity of the system.
[10] D_{SEM} – average particle size calculated by the SEM ± standard deviation.
