# Supplementary figures and images for: Effects of Modified Magnetite Nanoparticles on Bacterial Cells and Enzyme Reactions
Source: Nanomaterials (Basel). 2020 Jul 30;10(8):1499. doi: 10.3390/nano10081499 (PMC7466415; doi:10.3390/nano10081499)

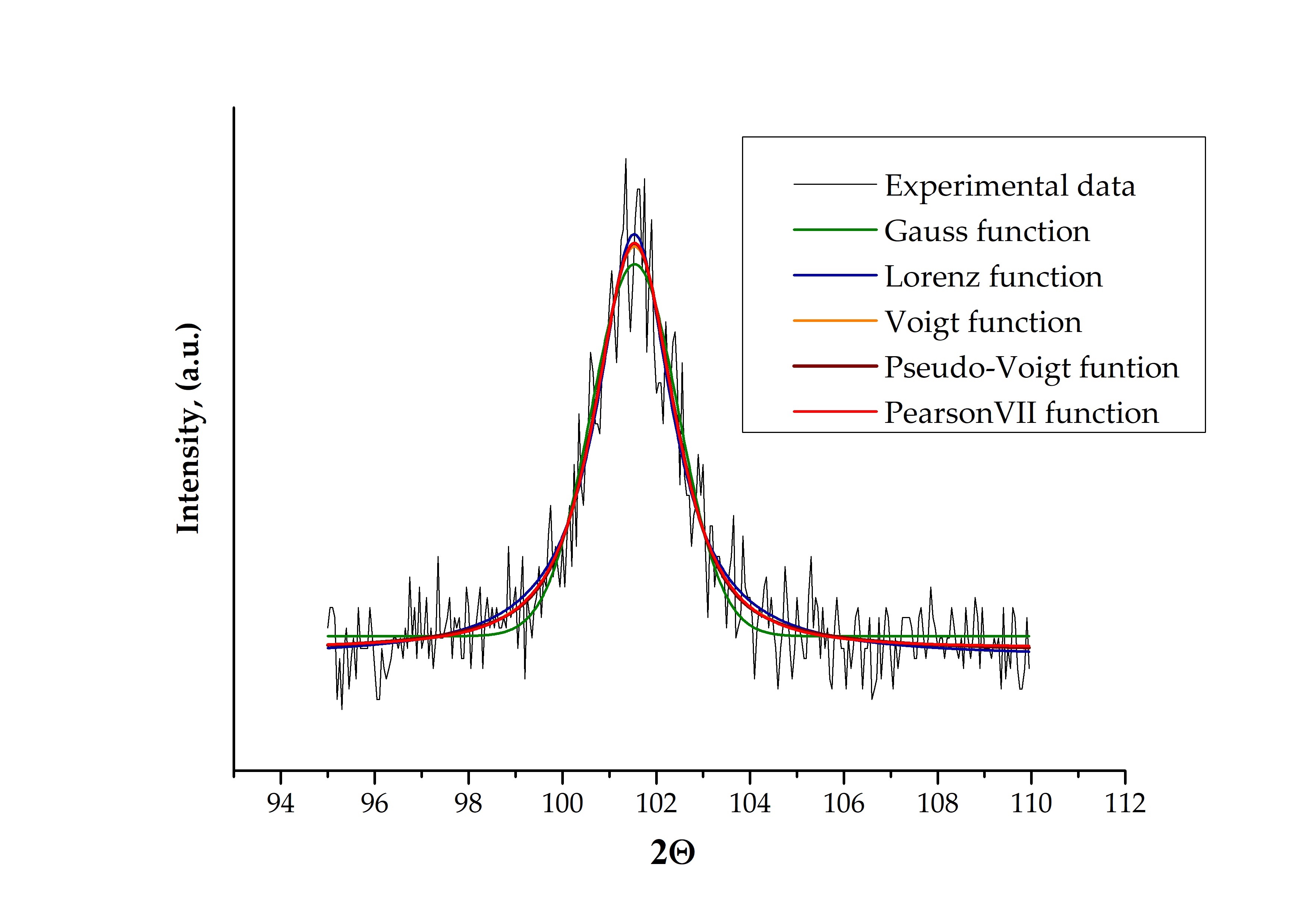

Supplement: Supplementary file 1 [file nanomaterials-10-01499-s001.zip › Supplementary files/Figure S1.jpg]
