# Supplementary material for: Effects of Modified Magnetite Nanoparticles on Bacterial Cells and Enzyme Reactions
Source: Nanomaterials (Basel). 2020 Jul 30;10(8):1499. doi: 10.3390/nano10081499 (PMC7466415; doi:10.3390/nano10081499)
Supplement: Supplementary file 1 [file Table S5.pdf › Supplementary files/Table S5.pdf]

Table S5. Statistics of relative bioluminescence induction period in 1,4-benzoquinone solution (Figure 8)

Samples	p
	**Concentrations**
Fe_3O_4	>0.05
Fe_3O_4/APTES	0.01
Fe_3O_4/HA	0.0001
	**Comparison by sample pairs**
Fe_3O_4/APTES- Fe_3O_4	0.01
Fe_3O_4/HA- Fe_3O_4	0.01
Fe_3O_4/HA- Fe_3O_4/APTES	>0.05

Bioactivity in relation to control:

Samples	Concentrations, mg/L												
Fe_3O_4-control	4.7E-12	4.7E-11	4.E-10	4.7E-9	4.E-8	4.E-7	4.E-6	4.E-5	4.E-4	4.7E-3	4.7E-2	4.7E-1	4.7E+0
	0.01	0.01	>0.05	>0.05	>0.05	>0.05	>0.05	>0.05	>0.05	>0.05	>0.05	>0.05	>0.05
	>0.05												
Fe_3O_4/APTES-control	1.1E-12	1.1E-11	1E-10	1E-9	1E-8	1E-7	1E-6	1E-5	1E-4	1.1E-3	1.1E-2	1.1E-1	1.1E+0
	>0.05	>0.05	0.01	0.01	>0.05	0.01	>0.05	>0.05	>0.05	>0.05	>0.05	>0.05	0.01
	0.01												
Fe_3O_4/HA-control	4.9E-13	4.9E-12	4.9E-11	4.9E-10	4.9E-9	4.9E-8	4.9E-7	4.9E-6	4.9E-5	4.9E-4	4.9E-3	4.9E-2	4.9E-1
	>0.05	>0.05	>0.05	>0.05	0.01	0.01	0.01	0.01	0.001	0.01	0.01	0.01	0.0001
	0.01												
